# Pan-cancer analysis of the prognostic and immunological role of PAQR4

**DOI:** 10.1038/s41598-022-25220-3

**Published:** 2022-12-08

**Authors:** Kaibin Wang, Jinhuan Meng, Xudong Wang, Mo Yan, Shuaibing Liu, Shaobo Yang, Shengxian Xu, Dongze Liu, Changying Li, Kuo Yang

**Affiliations:** Tianjin Institute of Urology, The Second Hospital of Tianjin Medical University, Tianjin, China

**Keywords:** Cancer, Immunology

## Abstract

Progestin and adipoQ receptor family member 4 (PAQR4) is a protein-coding gene. Recent studies have shown that PAQR4 is related to the development of multiple cancers. However, there is no systematic pan-cancer analysis of this gene. In this study, the expression of PAQR4, correlations with clinical prognosis, immune situation, and its potential molecular functions and mechanisms in pan-cancer were explored by bioinformatics analysis. The Cancer Genome Atlas was applied to investigate the relations between PAQR4 and clinical features, prognostic effects, and tumor immune microenvironment. R software was used to perform statistical analysis and figure creation. The expression of PAQR4 in BLCA and KIRC was validated by qRT-PCR and immunohistochemistry, and its function was explored by cellular experiments. Bioinformatics analysis revealed that PAQR4 was up-regulated in multiple cancers and related to poor prognosis. The high expression of PAQR4 was closely associated with high tumor stage, immune cell infiltration, tumor mutation burden, and microsatellite instability in different cancer types. In addition, the high expression of PAQR4 also indicated involvement in the immune regulatory pathways. The involvement of PAQR4 in the immune regulation of different tumors was confirmed by GSEA enrichment analysis. Moreover, PAQR4 was highly expressed in bladder cancer and renal clear cell carcinoma tissues and cell lines. Cell proliferation, migration, and invasion of bladder cancer and renal clear cell carcinoma cell lines were significantly decreased after the knockdown of PAQR4. This study elucidated the role of PAQR4 in carcinogenesis as well as tumor immunity. PAQR4 may serve as a potential prognostic biomarker in a variety of cancers.

## Introduction

As one of the leading causes of death, cancer poses a great threat to people's health and creates a great economic burden on society. Research indicates that there were an estimated 19.3 million new cancer cases and nearly 10 million cancer deaths worldwide in 2020. World Health Organization (WHO) estimated in 2019 that cancer is the first or second leading cause of death before age 70 in 112 of 183 countries, and the third or fourth leading cause in another 23 countries. Based on current incidence rates, an estimated 28.4 million new cancer cases will occur globally in 2040^1^. At present, there are no absolute cures for cancer. The majority of patients eventually die of cancer metastasis, recurrence, or complications. With the development of public databases, such as The Cancer Genome Atlas (TCGA), searching for specific molecular targets to improve cancer diagnosis and treatment has become more accessible.

PAQR4 belongs to a progesterone and lipid receptor family, which is a new family of membrane receptors consisting of 11 members (PAQR1 to PAQR11)^2,3^. PAQR4 has been shown to play a role in several types of cancer. In non-small cell lung cancer (NSCLC), PAQR4 was reported to promote tumor cell proliferation and metastasis, and also contribute to chemotherapy resistance^4,5^. PAQR4 has also been suggested to be involved in the carcinogenesis of breast cancer^6^ and the development of prostate cancer^7,8^, liver cancer ^9^, and gastric cancer ^10^.

At present, studies on PAQR4 mostly focused on a specific type of cancer. Our study is for the first time to analyze the role of PAQR4 across diverse cancer types. The expression of PAQR4, its correlations with clinical characteristics, prognosis, immune system, and its potential molecular functions and mechanisms in pan-cancer were systematically accessed through public databases. We further confirmed the up-regulated expression of PAQR4 in BLCA and KIRC tissues and its promotion of BLCA and KIRC. In summary, PAQR4 has the potential value as a biomarker for determining prognosis in a variety of cancers.

## Results

### Expression analysis of PAQR4

**Figure 1:**
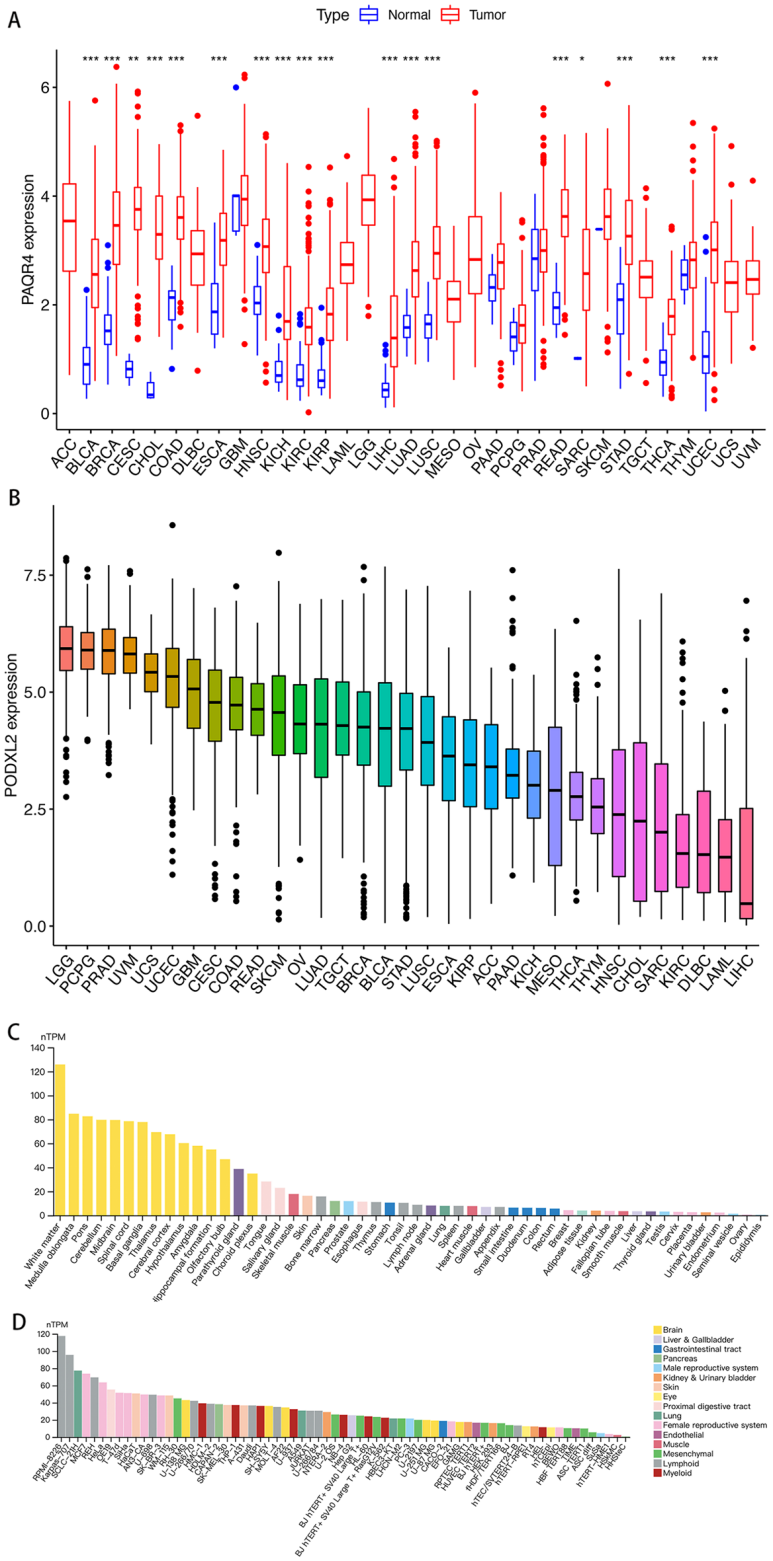
PAQR4 expression in tumors, normal tissues, and cell lines. (**A**) Differential expression of PAQR4 in tumors and normal tissues. Red represents tumors, blue represents normal tissues, *, **, *** represent *P-value* < 0.05, *P-value* < 0.01 and *P-value* < 0.001, respectively. (**B**) PAQR4 mRNA expression sorting in 33 cancers. (**C**) Overview of PAQR4 expression in cell lines in the HPA dataset. (**D**) Overview of PAQR4 mRNA expression in normal tissues based on the HPA dataset.

In the UCSC Xena database, compared with normal tissues, PAQR4 mRNA expression was significantly upregulated in 18 cancers, including BLCA, BRCA, CHOL, CAOD, ESCA, HNSC, KICH, KIRC, KIRP, LIHC, LUAD, LUSC, READ, STAD, THCA, UCEC, *P-value* <0.001; CESE, *P-value* <0.01; SARC, *P-value* <0.05 (Fig. 1A). The expression level of PAQR4 in 33 cancers was further sorted from high to low. As shown in Fig. 1B, the highest expression was found in GBM while the lowest was in LIHC. In the HPA database, PAQR4 expression among normal tissues was highest in white matter while lowest in epididymis (Fig. 1C). Besides, PAQR4 showed significantly high expression in the lymphoid cell lines RPMI-8226 and Karpas-707 (Fig. 1D).

**Figure 2:**
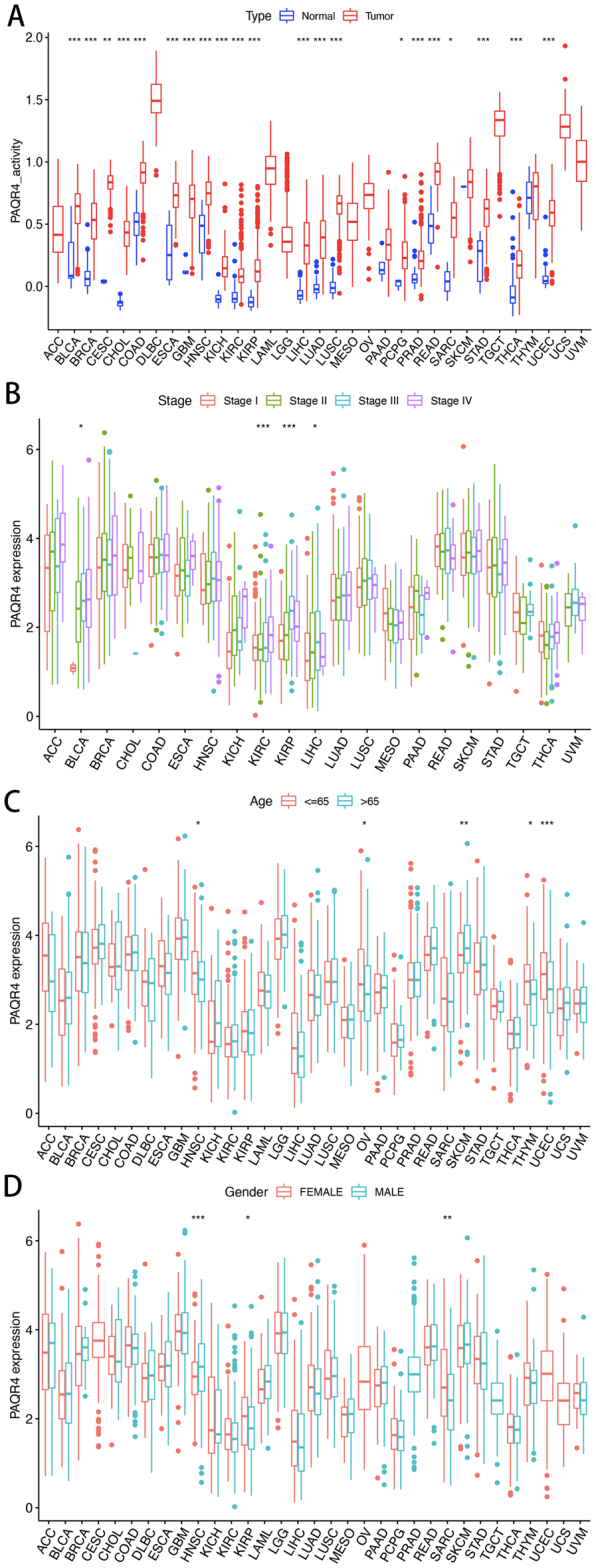
Activity and clinical characteristics of PAQR4 in pan-cancer. (**A**) Differences in PAQR4 expression activity in tumors and normal tissues. (**B**–**D**) Correlation of PAQR4 expression with tumor stage, age, and gender in pan-cancer. *, **, *** represent *P-value* < 0.05, *P-value* < 0.01 and *P-value* < 0.001, respectively.

As shown in Fig. 2A, the activity of PAQR4 in cancer tissues was generally higher than that in normal tissues. High levels of PAQR4 in BLCA, KIRC, KIRP, and LIHC were associated with relatively poorer tumor stages (BLCA, LIHC, *P-value* <0.05; KIRC, KIRP, *P-value* <0.001) (Fig. 2B). Meanwhile, high expression of PAQR4 in HNSC, OV, THYM, and UCEC was found in patients not older than 65 years, but the opposite in SKCM (*P-value* <0.05) (Fig. 2C). In addition, PAQR4 expression levels were higher in females in KIRP and SARC, while higher in males in HNSC (*P-value* <0.05) (Fig. 2D).

### PAQR4 expression and patient’s prognosis

**Figure 3:**
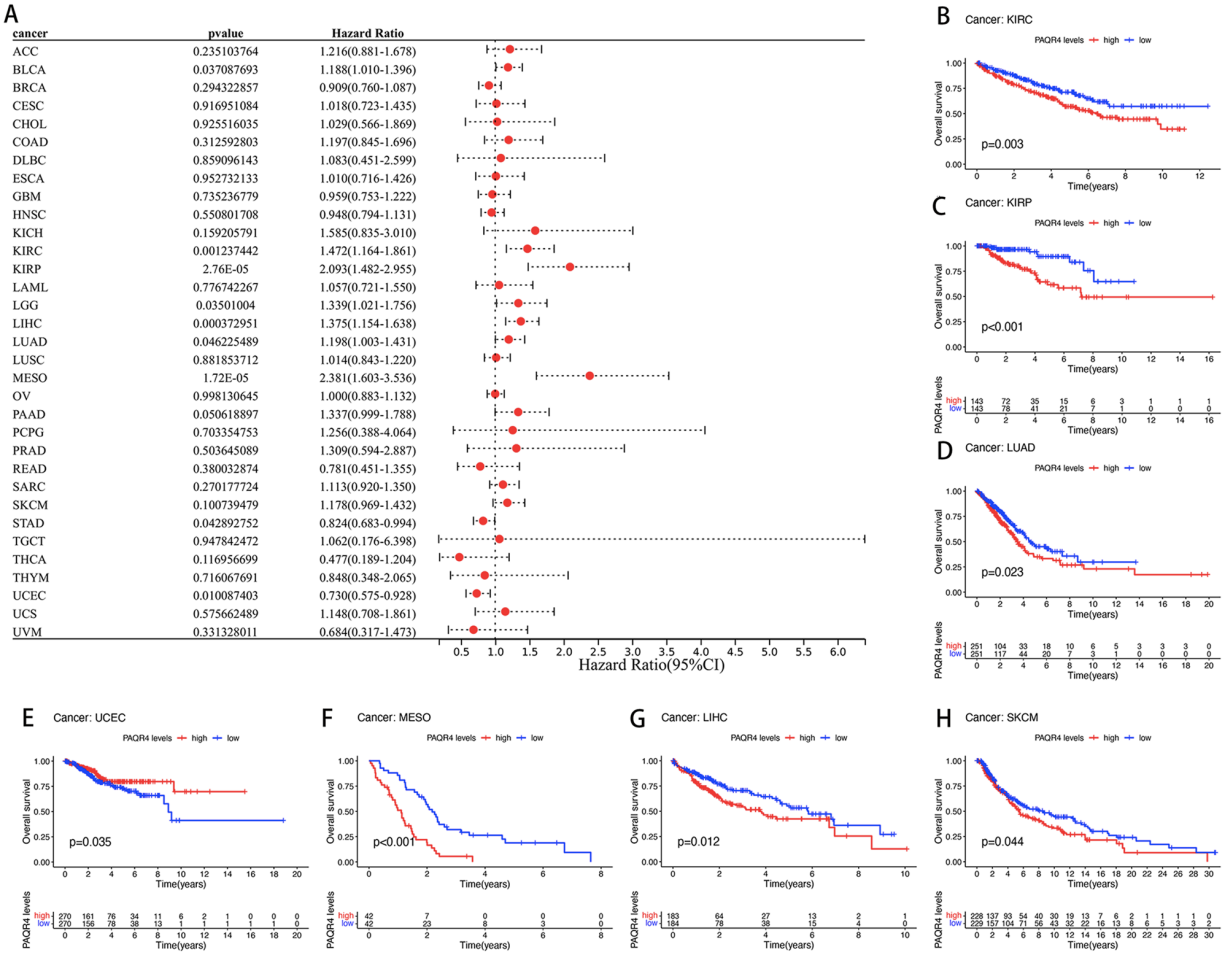

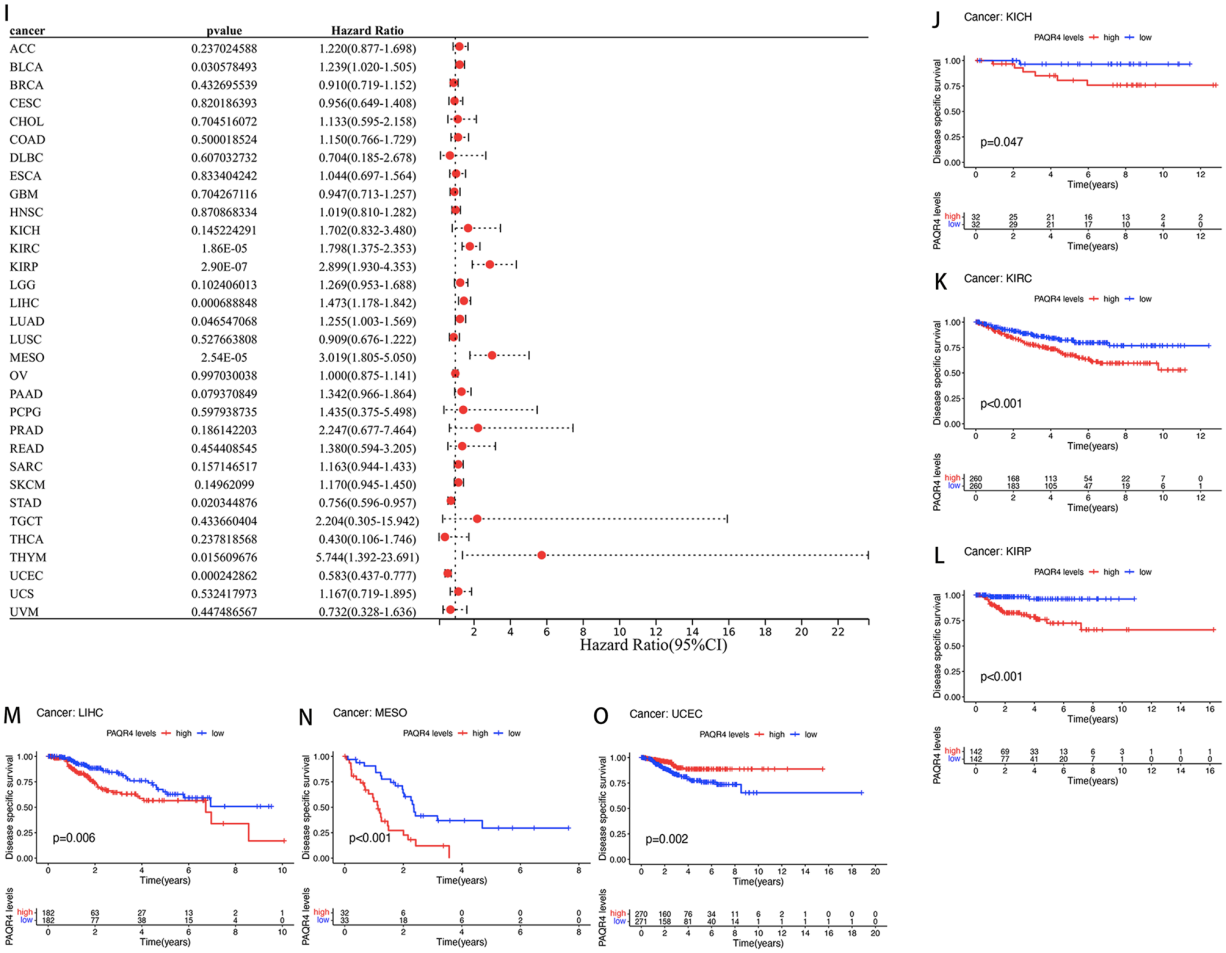
Prognostic value of PAQR4 in pan-cancer. (**A**) Forest plot of the association between PAQR4 and overall survival (OS) in 33 tumors. (**B**–**H**) Kaplan–Meier analysis of the association between PAQR4 expression and OS in each tumor. (**I**) Forest plot of the association between PAQR4 and disease-specific survival (DSS) in 32 tumors. (**J**–**O**) Kaplan–Meier analysis of the association between PAQR4 expression and DSS.

The prognostic value of PAQR4 in pan-cancer was assessed by analyzing transcriptomic data and clinical data from 33 cancers acquired by UCSC Xena. The relation between PAQR4 and OS was demonstrated by Cox analysis. The resultant elevated PAQR4 expression implied a higher risk in BLCA (*HR*=1.188, *P-value* <0.05), KIRC (*HR*=1.472, *P-value* <0.01), KIRP (*HR*=2.093, *P-value* <0.001), LGG (*HR*=1.339, *P-value* <0.05), LIHC (*HR*=1.375, *P-value* <0.001), LUAD (*HR*=1.198, *P-value* <0.05), MESO (*HR*=2.381, *P-value* <0.001), while in STAD (*HR*=0.824, *P-value* <0.05) and UCEC (*HR*=0.730, *P-value* <0.05) it implied a lower risk (Fig. 3A). Kaplan-Meier survival analysis suggested an association between PAQR4 expression and OS, with patients in the high expression group in KIRC (*P-value* <0.001), KIRP (*P-value* <0.001), LIHC (*P-value* <0.05), LUAD (*P-value* <0.05), MESO (*P-value* <0.001), and SKCM (*P-value* <0.05) having the poorer OS, while in UCEC (*P-value* <0.05) it suggested a better prognosis (Fig. 3B-H).

The study also analyzed the prognostic value of PAQR4 in disease-specific survival (DSS), disease-free survival (DFS), and progression-free survival (PFS) in tumor patients. The high expression of PAQR4 showed poor DSS in Cox analysis of BLCA (*HR*=1.239, *P-value* <0.05), KIRC (*HR*=1.798, *P-value* <0.001), KIRP (*HR*=2.899, *P-value* <0.001), LIHC (*HR*=1.473, *P-value* <0.001), LUAD (*HR*=1.225, *P-value* <0.005), MESO (*HR*=3.019, *P-value* <0.001), THYM (*HR*=5.744, *P-value* <0.05) (Fig. 3I) and KM analysis of KICH, KIRC, KIRP, LIHC, MESO (all *P-value* <0.05; Fig. 3J-N), while in Cox analysis of UCEC (*HR*=0.583, *P-value* <0.001), STAD (*HR*=0.756, *P-value* <0.05) (Fig. 3I) and KM analysis of UCEC (*P-value* <0.01; Fig. 3O) showed better DSS.

In addition, the high expression of PAQR4 in Cox analysis of CESC (*HR*=1.915, *P-value* <0.05), LIHC (*HR*=1.263, *P-value* <0.01), SARC (*HR*=1.299, *P-value* <0.05), THCA (*HR*=2.192, *P-value* <0.05) ( Supplementary Fig. 1A) and KM analysis of LIHC (*P-value* <0.05), THCA (*P-value* <0.01) ( Supplementary Fig. 1C,D) predicted a shorter DFS time, while in KM analysis of ESCA (*P-value* <0.05) ( Supplementary Fig. 1B), UCEC (*P-value* <0.05) ( Supplementary Fig. 1E) predicted a longer DFS time. Finally, for PFS, high expression of PAQR4 showed a poorer prognosis in Cox analysis of CESC (*HR*=1.469, *P-value* <0.05), KIRC (*HR*=1.606, *P-value* <0.001), KIRP (*HR*=1.828, *P-value* <0.001), LGG (*HR*=1.275, *P-value* <0.05), LIHC (*HR*=1.294, *P-value* <0.001), MESO (*HR*=1.753, *P-value* <0.01), SARC (*HR*=1.243, *P-value* <0.05)( Supplementary Fig. 1F) as well as KM analysis of KIRC, KIRP, LIHC, MESO, SARC, THCA (all *P-value* <0.05; Supplementary Fig. 1H-M), while in Cox analysis of UCEC (*HR*=0.795, *P-value* <0.05), STAD (*HR*=0.807, *P-value* <0.05) ( Supplementary Fig. 1F), as well as KM analysis of GBM (*P-value* <0.05; Supplementary Fig. 1G), UCEC (*P-value* <0.05; Supplementary Fig. 1N), showed better prognosis.

### PAQR4 expression and tumor immunity

The above study examined the association between PAQR4 expression and prognosis in pan-cancer, and the relation between the expression of PAQR4 and tumor immunity was investigated next. PAQR4 expression was negatively correlated with both immune score and stromal score in GBM and PAAD tumors. PAQR4 expression was negatively correlated with the immune score in ACC and TGCT, it was also negatively correlated with the stromal score in THYM (Supplementary Fig. 2).

**Figure 4:**
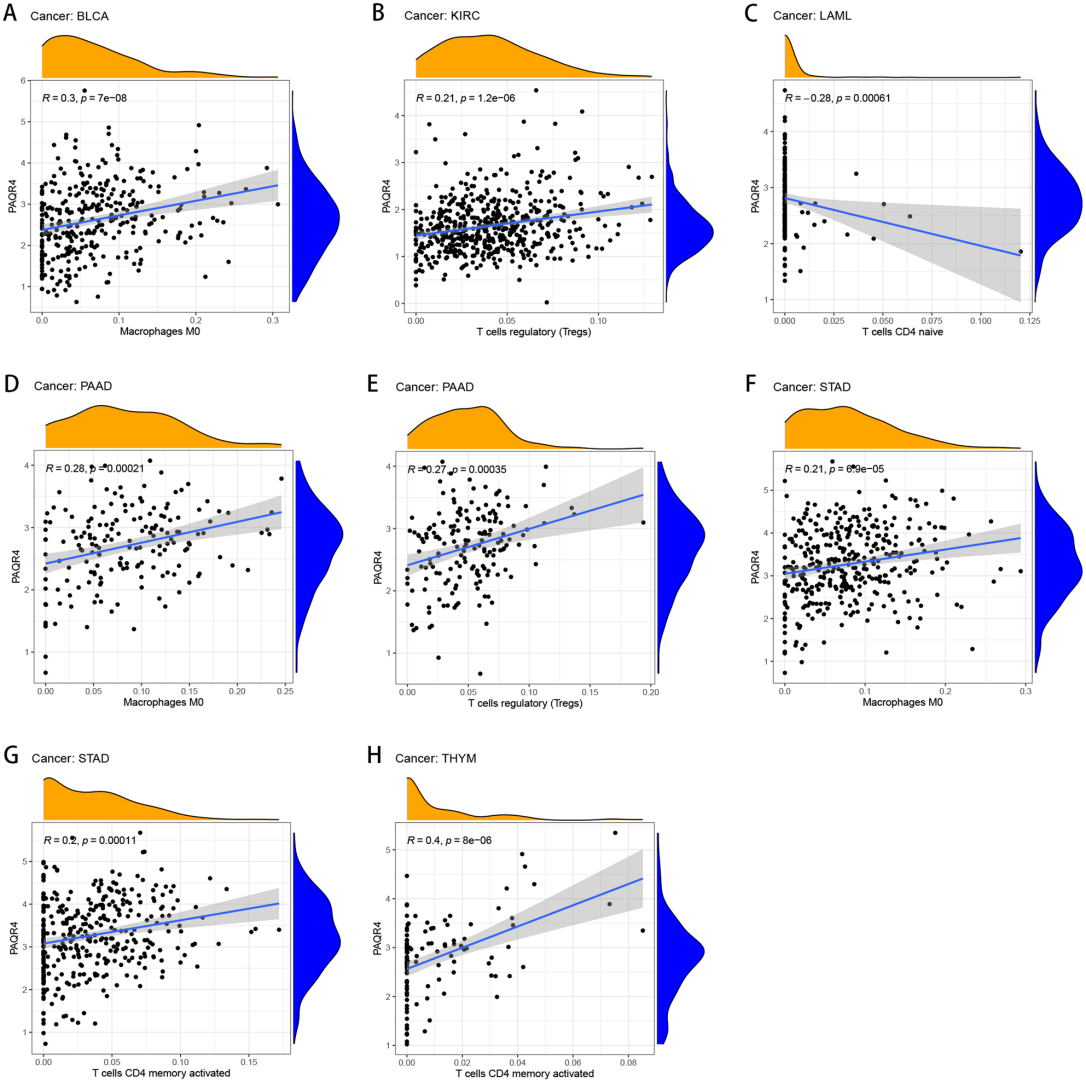
Association of PAQR4 expression with immune cell infiltration in a variety of tumors. (**A**–**H**) Correlation of PAQR4 expression with immune cell infiltration in BLCA, KIRC, LAML, PAAD, STAD, and THYM.

The relationship between immune cell infiltration and PAQR4 expression was also explored. PAQR4 expression was positively correlated with Macrophages M0 in BLCA, PAAD, and STAD, T cell regulation (Tregs) in KIRC and PAAD, T cells CD4 memory activated in STAD and THYM, while negatively correlated with T cells CD4 naive in LAML (Fig. 4). More results about the association of PAQR4 expression with other immune cell infiltration in tumors were shown in Supplementary Fig. 3. These findings strongly suggested the potential of PAQR4 to modulate the tumor microenvironment.

**Figure 5:**
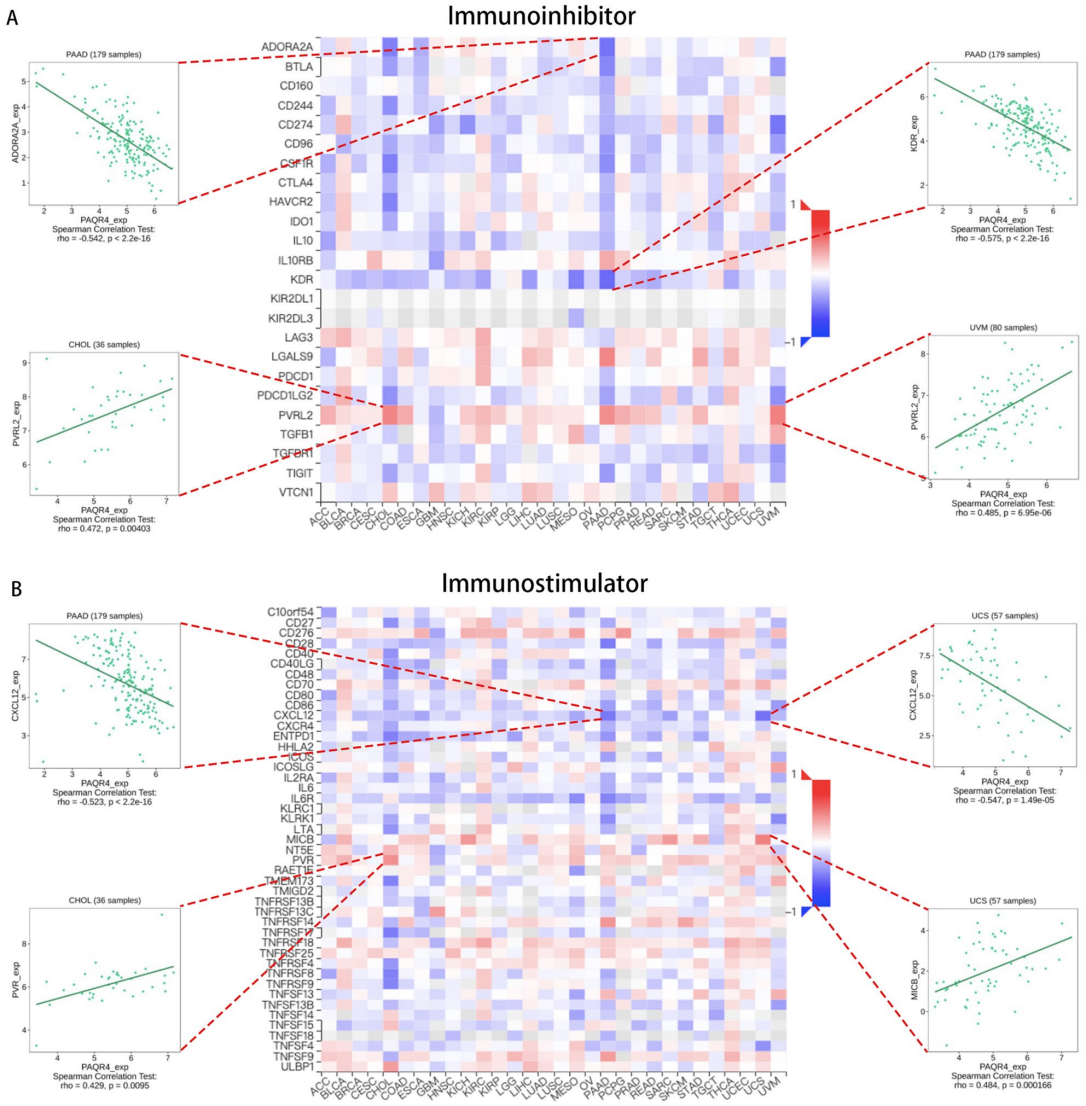

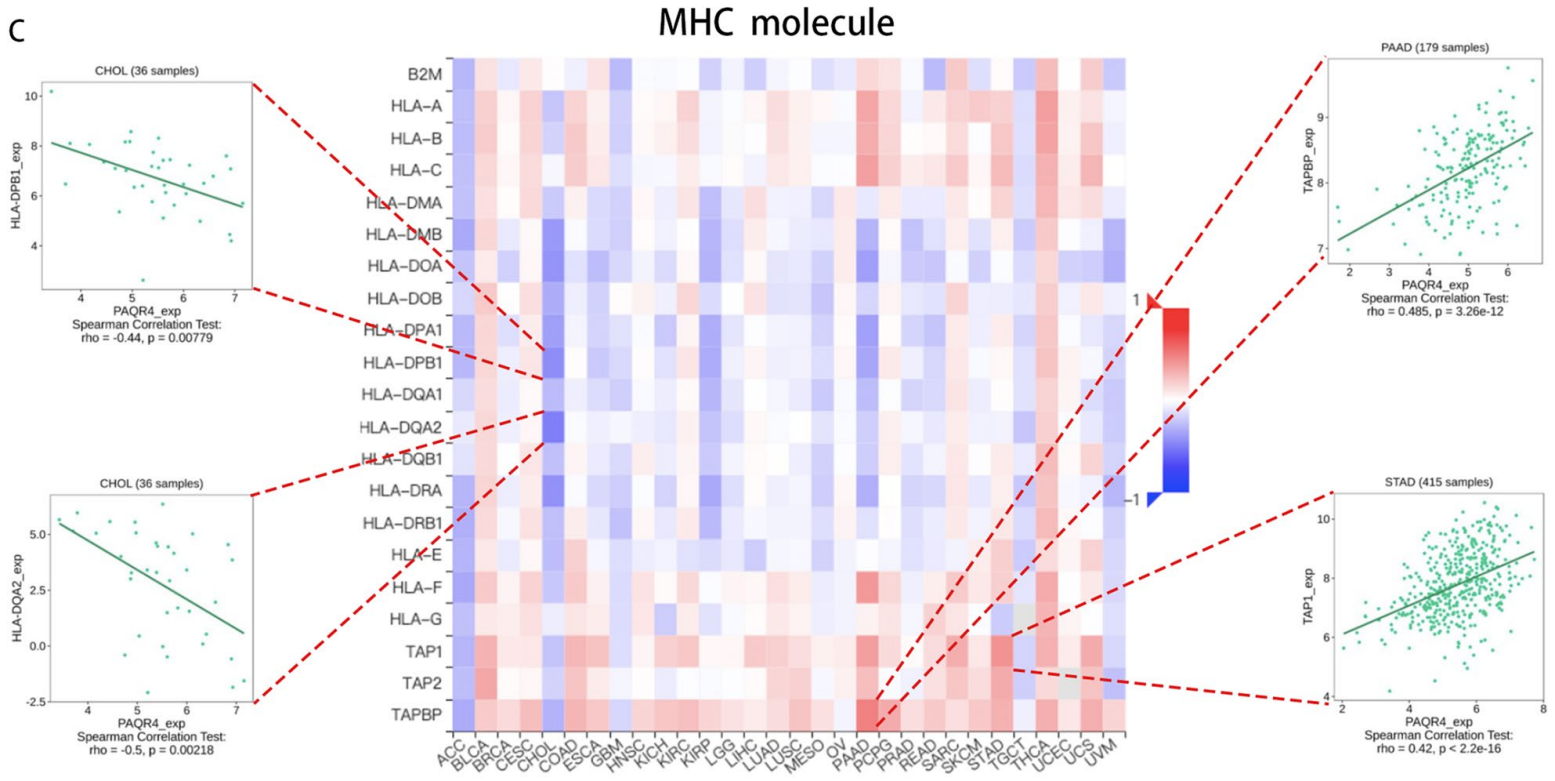
Analysis of the relationship between three immunomodulators and PAQR4 expression in pan-cancer based on TISIDB. (**A**) Correlation of PAQR4 expression with immunoinhibitors. (**B**) Correlation of PAQR4 expression with immunostimulators. (**C**) Correlation of PAQR4 with the expression of MHC molecules. The immune genes most strongly correlated with PAQR4 were drawn scatter plots.

In addition, the relations between the expression of PAQR4 and immunoinhibitors, immunostimulators, and MHC molecules in pan-cancer were analyzed by the TISIDB database. Figure 5A showed the heat map of 24 immunoinhibitors in correlation with the expression of PAQR4. PAQR4 expression was negatively linked to ADORA2A in PAAD (R<-0.5, *P-value* <0.05), and it was also negatively linked to KDR in PAAD (R<-0.5, *P-value* <0.05). We also found that PAQR4 was positively linked to PVRL2 in CHOL and UVM (R>0.4, *P-value* <0.05). Figure 5B showed the relation of PAQR4 with 45 types of immune stimulators. The correlation of PAQR4 expression with MHC molecules was also analyzed based on the TISIDB database (Fig. 5C).

The association of PAQR4 expression with immune subtypes of multiple cancer types was summarized through the TISIDB database. All tumor samples were classified into six immunological subtypes: C1 (wound healing); C2 (IFN-gamma dominant); C3 (inflammatory); C4 (lymphocyte depleted); C5 (immunologically quiet); C6 (TGF-b dominant). The complete results were shown in Supplementary Fig. 4.

### PAQR4 and tumor mutation burden, tumor microsatellite instability

**Figure 6:**
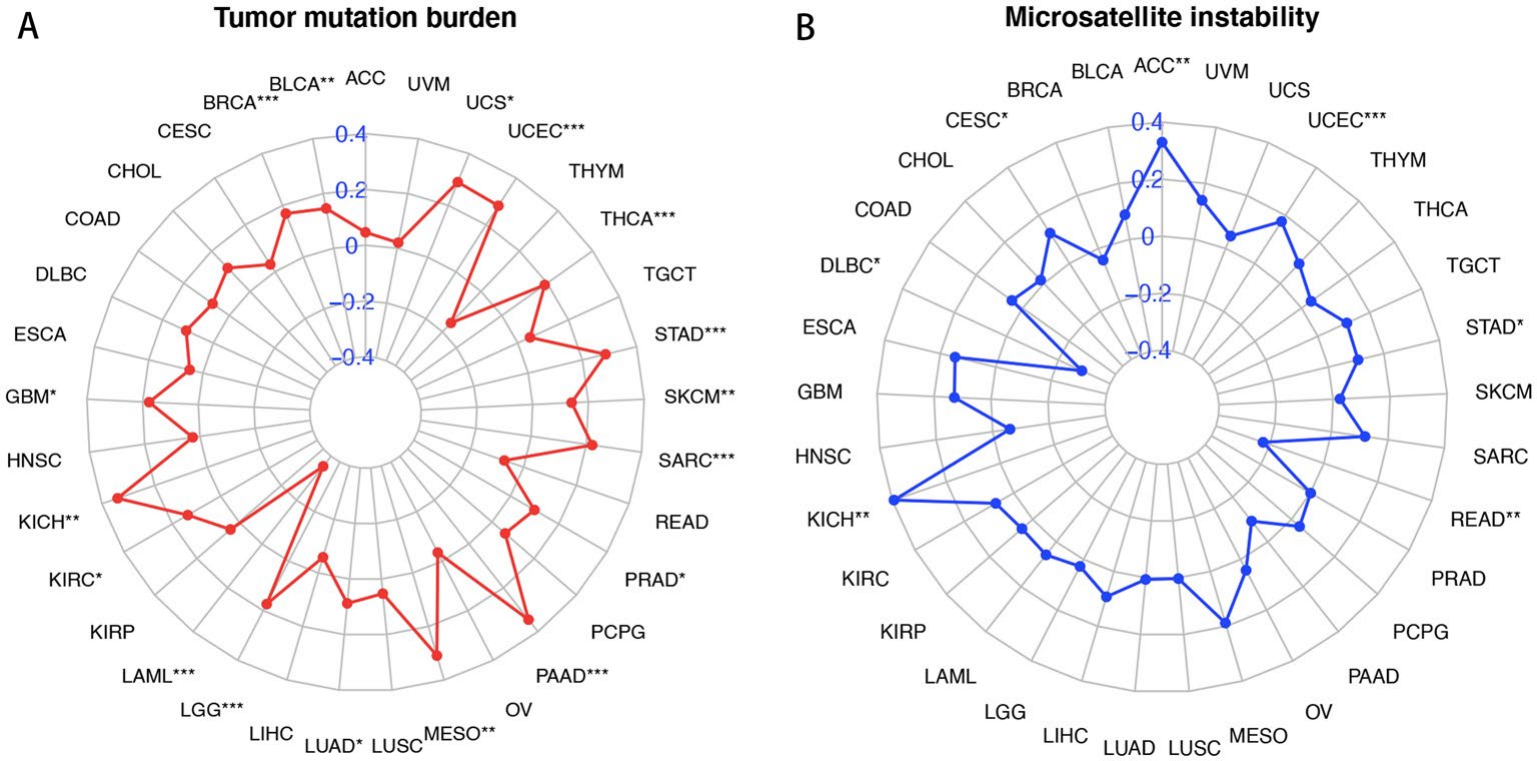
Correlation of PAQR4 with tumor mutation burden and microsatellite instability. Association of PAQR4 expression with tumor mutation burden (**A**), microsatellite instability (**B**). *, **, *** represent *P-value* < 0.05, *P-value* < 0.01 and *P-value* < 0.001, respectively.

Tumor mutation burden has been reported to be able to predict survival after immunotherapy across multiple cancer types and higher TMB may predict greater clinical benefit ^11^. MSI status may predict cancer response or resistance to certain chemotherapies^12^. We analyzed the association of PAQR4 expression with TMB and MSI in pan-cancer. PAQR4 was positively correlated with TMB in BLCA, BRCA, GBM, KICH, KIRC, LGG, LUAD, MESO, PAAD, PRAD, SARC, SKCM, STAD, THCA, UCEC, and UCS, while negatively in LAML (Fig. 6A). PAQR4 was positively correlated with MSI in the ACC, CESC, KICH, STAD, and UCEC cohorts, while negatively in DLBC and READ (Fig. 6B). These findings strongly suggested the potential value of PAQR4 in cancer therapy.

### GSEA enrichment analysis

**Figure 7:**
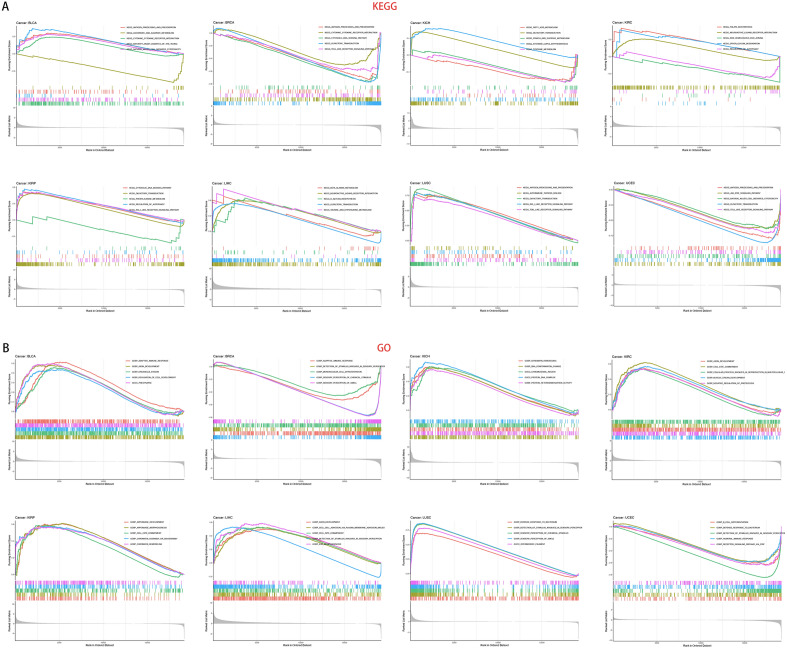
GSEA enrichment analysis. (**A**) Analysis of KEGG pathway of PAQR4 in pan-cancer. (**B**) Analysis of GO function of PAQR4 in pan-cancer. The peak of the rising curve represented the enriched pathway and function in the high PAQR4 expression group, and the peak of the falling curve represented the enriched pathway and function in the low PAQR4 expression group.

GSEA enrichment analysis was performed to investigate the biological functions of PAQR4 in a variety of tumors. Eight cancers with significant PAQR4 overexpression and prognostic significance were selected to summarize their KEGG pathway analysis^13–15^ and GO functional annotation results (Fig. 7). PAQR4 positively regulated organelle division, formation, and maturation of cells in BLCA, and positively regulated cell differentiation, dynamic changes in chromatin during the cell division cycle, and disruption of chromosome structure in KICH, KIRC, and KIRP tumors, etc. PAQR4 also positively regulated cell adhesion in LIHC. The results of the GSEA analysis also showed that a large number of tumors involved the sensory perception of chemical stimuli. And PAQR4 generally played a positive regulatory role in these pathways. The antigen processing and presentation pathway was activated in BLCA, BRCA, LUSC, and USEC, however, it appeared in the high PAQR4 expression group in BLCA and LUSC, while appeared in the low PAQR4 expression group in BRCA and USEC. In addition, cytokine-cytokine receptor interactions, Toll-like receptor signaling pathways, autophagy regulation, metabolism, and other related pathways were involved in many tumors.

### PAQR4 was highly expressed in cancer tissues, while knockdown of PAQR4 inhibited the proliferation of cancer cell lines

**Figure 8:**
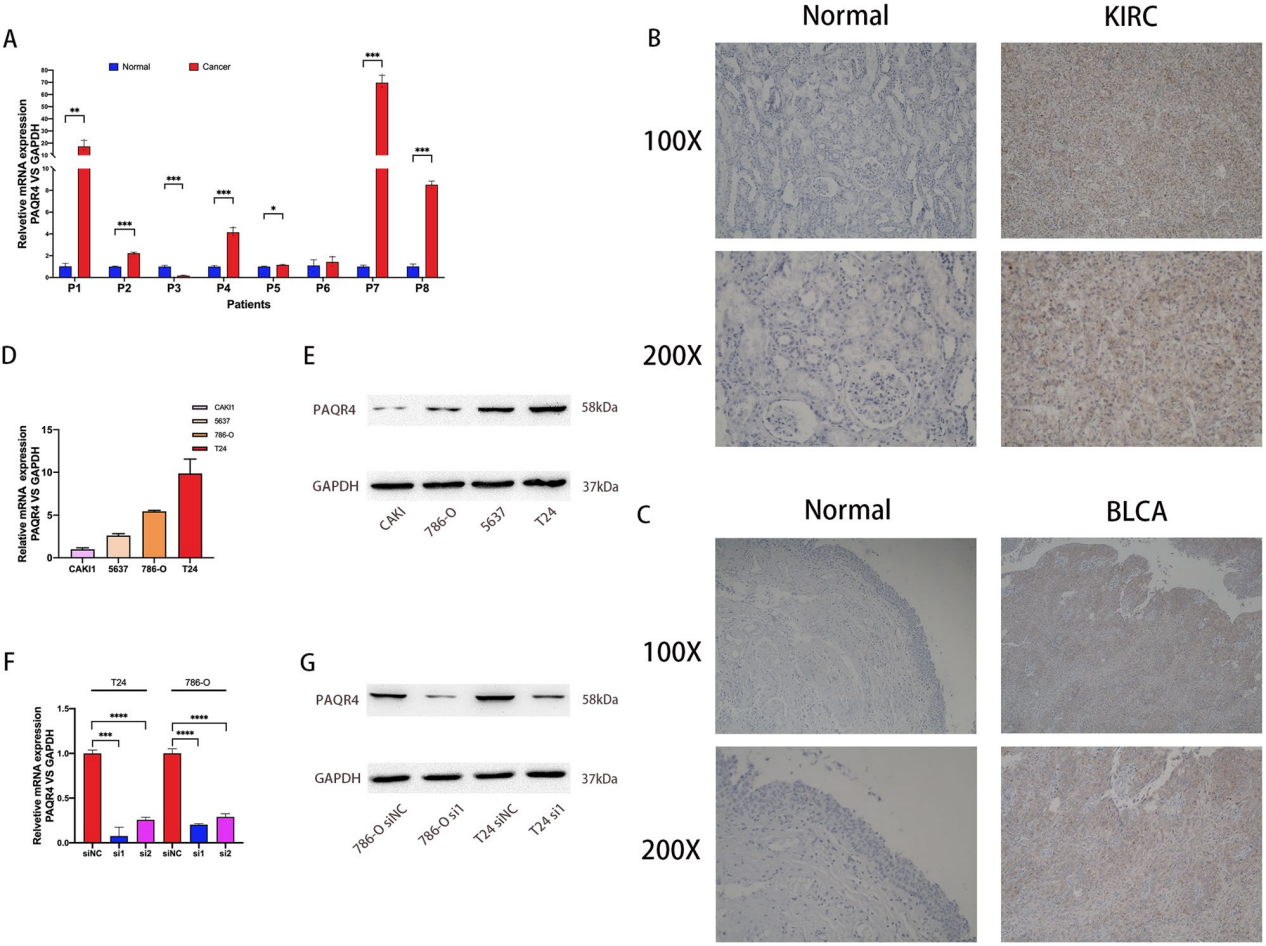
Expression of PAQR4 in cancer tissues and cancer cell lines. (**A**) The mRNA expression of PAQR4 in BLCA tissues. (**B**) Immunohistochemistry: Negative for PAQR4 in normal kidney tissue; Renal clear cell carcinoma tissue positive for PAQR4. (**C**) Immunohistochemistry: Negative for PAQR4 in normal bladder tissue; Bladder cancer tissue positive for PAQR4. (**D**–**E**) The mRNA and protein expression of PAQR4 in bladder cancer cell lines 5637, T24, and renal clear cell carcinoma cell lines CAKI1, 786-O.(**F**–**G**) Knockdown efficacy in T24 and 786-O cells. All experiments were repeated at least three times. *, **, ***, **** represent *P-value* < 0.05, *P-value* < 0.01, *P-value* < 0.001, and *P-value* < 0.0001, respectively.

**Figure 9:**
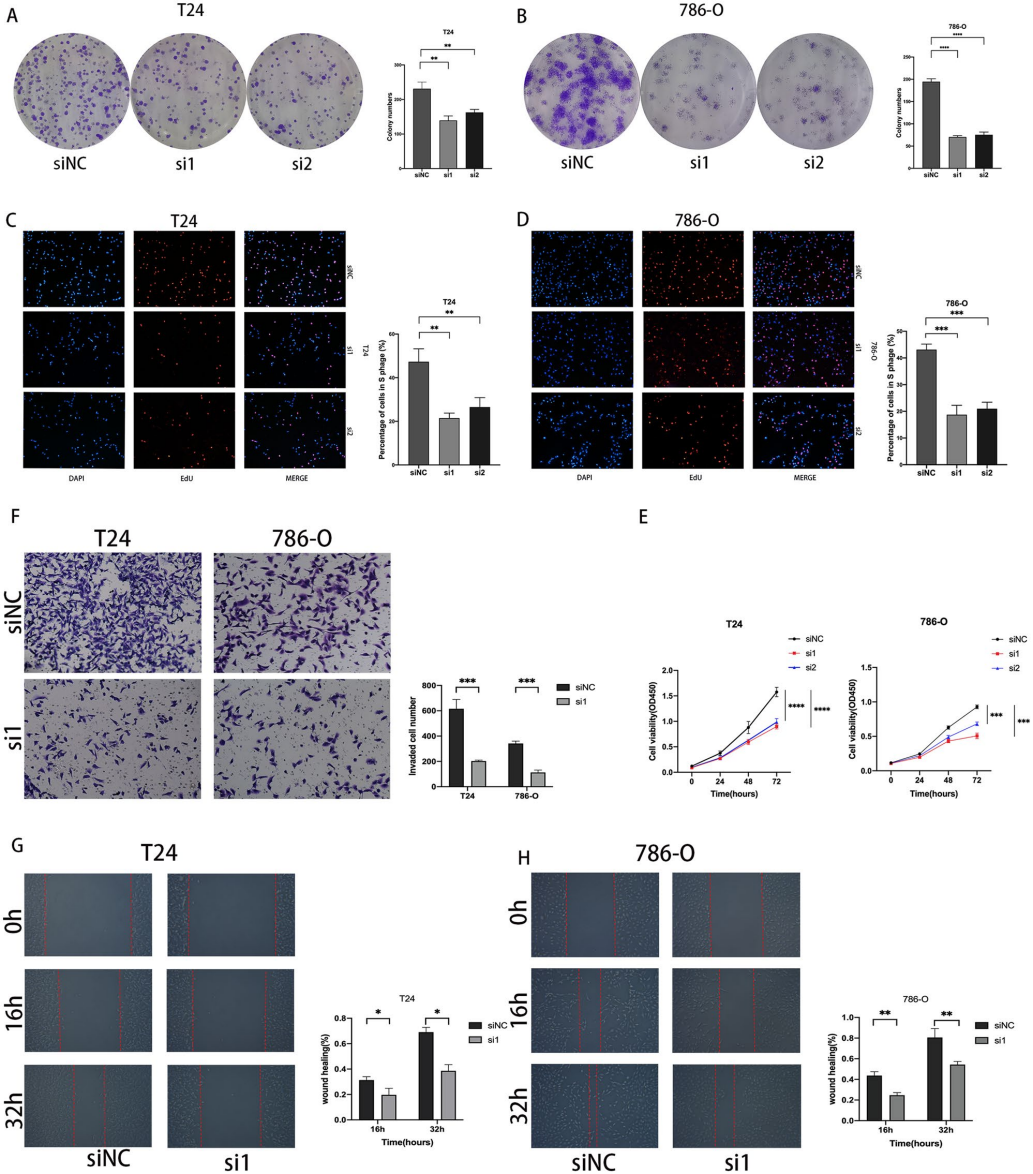
PAQR4 promotes the proliferation, invasion, and migration ability of cancer cells. (**A**, **B**) Knockdown of PAQR4 inhibited T24 and 786-O cells colony formation ability. (**C**, **D**) Knockdown of PAQR4 inhibited T24 and 786-O cells proliferation examined by EdU assay. (**E**) Knockdown of PAQR4 inhibited T24 and 786-O cells proliferation examined by CCK-8 assay. (**F**) Transwell invasion assays were performed in T24 and 786-O cells. (**G**, **H**) Knockdown of PAQR4 inhibited T24 and 786-O cells migration. All experiments were repeated at least three times. *, **, ***, **** represent *P-value* < 0.05, *P-value* < 0.01, *P-value* < 0.001, and *P-value* < 0.0001, respectively.

QRT-PCR results showed that PAQR4 mRNA level was significantly higher in bladder cancer tissues (75%) than in adjacent normal tissues (*P-value*<0.05) (Fig. 8A). The results of immunohistochemistry showed that PAQR4 was highly expressed in cancer tissues of patients with bladder cancer and renal clear cell carcinoma (Fig. 8B,C). Bladder cancer cell lines T24 and renal clear cell carcinoma cell line 786-O cells with high levels of PAQR4 mRNA and protein expression (Fig. 8D,E) were further selected to be transfected with siRNA to target PAQR4, and the knockdown efficiency was verified by qRT-PCR and western blot (Fig. 8F,G).

the The results of the colony formation assay showed that the knockdown of PAQR4 significantly reduced the colony formation ability of T24 and 786-O compared with siNC (Fig. 9A,B). EdU experiments showed consistent results (Fig. 9C,D). CCK-8 results showed that the proliferative activity was significantly decreased after the knockdown of PAQR4 (Fig. 9E). At the same time, the decreased invasive ability of cancer cells was confirmed by the transwell experiment (Fig. 9F). The wound healing assay results showed that the migration ability of T24 and 786-O decreased after the knockdown of PAQR4 (Fig. 9G-H).

## Discussion

PAQR family contains eleven members, and previous studies have focused more on PAQR3, a novel tumor suppressor gene that is downregulated in different types of human cancers. PAQR4 is a close homolog of PAQR3, which has been shown to have an opposite effect on tumors in the available studies^2,3^.

In this study, PAQR4 expression was unambiguously upregulated in 18 cancers, which was consistent with the results of existing studies on individual cancers. Ten of the eighteen tumors which have high PAQR4 expression showed a correlation with poor prognosis, especially BLCA, KIRC, KIRP, and LIHC. Although MESO was not classified in the group of high PAQR4 expression because of the lack of normal controls, high PAQR4 expression in MESO still suggested a significantly poorer prognosis. Remarkably, the expression of PAQR4 increased with the progression of tumor stage in BLCA, KIRC, KIRP, and LIHC. All these results strongly suggested that PAQR4 might act as a risk factor for tumor progression.

So far, little is known about the potential function and mechanism of PAQR4 in tumors. PAQR4 has been shown to promote cell proliferation and metastasis of NSCLC. Our cytological experiments confirmed this effect on BLCA and KIRC cells. Xu’s group revealed that increased expression of PAQR4 in NSCLC enhanced *in vivo* xenograft tumor formation through inhibiting Nrf2 protein^4^. Lu et al. found that PAQR4 could promote the progression of HCC by activating the PI3K/AKT pathway^9^. PAQR4-mediated PI3K/Akt pathway was also reported to increase tumorigenesis and metastasis in prostate cancer. CDK4 might be another key factor in PAQR4-mediated tumor progression. Studies in NSCLC^6^ and breast cancer suggested PAQR4 developed a tumorigenic effect by regulating the ubiquitination and degradation of CDK4^16^. The potential functions and mechanisms of PAQR4 in tumors still need to be investigated.

Since the tumor immune microenvironment (TIME) which could be influenced by multiple factors has been gradually recognized as a key contributor to cancer progression, we were full of curiosity about the relevance of PAQR4 and tumor immunity. Our analysis found a correlation between immune cell infiltration and PAQR4 expression in a variety of tumors. Macrophages M0 positively correlated with PAQR4 expression in tumors, it has been documented that Macrophages M0 polarized to M2 type macrophages promote tumor progression^17^. The literature showed that T cell regulation (Tregs) promoted cancer progression by limiting anti-tumor immunity and promoting angiogenesis^18–20^. Our analysis suggested that PAQR4 expression in KIRC, PAAD was positively correlated with T cell regulation (Tregs). The poor prognosis of PAQR4 high-expression tumor patients might be due to anti-tumor immunity restricted by the enrichment of Treg and Mo macrophages.

Analysis of the GSEA enrichment results for PAQR4 showed that in pan-cancer, PAQR4 high expression was commonly enriched in antigen processing and presentation pathway. In BLCA, KIRP, LUSC, and MESO, we observed that PAQR4 high expression was enriched in this pathway, and PAQR4 high expression in all these cancers suggested a poor prognosis. Numerous studies have shown this pathway played an important role in cancer. It was associated with the tumor's immune escape^21,22^. Combined with the above relation of PAQR4 in tumors with macrophages, T cells, etc., we hypothesized that PAQR4 might influence tumor progression by affecting tumor immunity, but the mechanisms and the key immune cells needed to be explored in subsequent studies.

We also found that the Toll-like signaling pathway was also enriched in the PAQR4 high expression group. Previous studies have suggested that this pathway might be associated with innate immunity and might play a role in enhancing antigen presentation^23^. These suggested the potential value of exploring the relationship between PAQR4 and tumor immunity.

In the correlation analysis of TMB and MSI, the vast majority of tumor TMB and MSI were positively correlated with PAQR4 expression, suggesting that immunotherapy and chemotherapy targeting PAQR4 in cancer treatment might have great significance. Our study also showed that PAQR4 had the potential value as a marker to differentiate tumor immune subtypes and guide precision therapy. We analyzed the relation between PAQR4 and three immunomodulators in pan-cancer using the TISIDB database. The correlation between each immunomodulator and PAQR4 can be visualized through heat maps, providing ideas for subsequent studies. All of these suggested that PAQR4 might be a potential therapeutic target and there was the possibility of finding immunotherapy modulators.

Interestingly, we found that although UCEC exhibited PAQR4 upregulation, all survival analyses showed that high expression of PAQR4 instead suggested lower risk. And PAQR4 was more significantly upregulated in UCEC in patients no older than 65 years, suggesting to us that PAQR4 might emerge as a protective factor in younger women of UCEC. At the same time, in the related GSEA analysis, PAQR4 low expression was enriched in the antigen processing and presentation pathway and Toll-like signaling pathway.

Based on the above studies, we hypothesized that PAQR4 might cause poor prognosis in most tumors by blocking the infiltration of anti-tumor immune cells. In young women with UCEC, hormonal effects might interfere with this process and affect patient prognosis. The related mechanism needed to be further investigated.

Our study systematically analyzed the relationship between PAQR4 and pan-cancer and the role of immunity. The function of PAQR4 in BLCA and KIRC cancer cell lines was briefly verified by cellular experiments for the first time. Although PAQR4 is highly expressed in many cancers, the research on PAQR4 is still limited, and the detailed mechanism of the role of PAQR4 in cancer has not been elaborated yet. Although our study has analyzed and explored this content, it was only based on fewer existing studies as well as bioinformatics analysis and lacked experimental validation, which is the focus of our next study. In addition, we have mainly conducted in vitro experiments for validation, and in vivo experiments need to be conducted later. The number of clinical samples was too small to perform clinical information analysis. In conclusion, PAQR4 showed its potential as a diagnostic and prognostic marker and deserves further investigation.

## Methods

### Data collection

Transcriptome data and related clinical data of 33 cancers were downloaded from TCGA through the UCSC Xena platform (https://xenabrowser.net/datapages/). The expression information of PAQR4 in normal tissues and cell lines was obtained from the website of Human Protein Atlas (http://www.proteinatlas.org/).

### Differential expression analysis and gene activity

We used the “limma^24^ (3.50.3)” package of R software (version 4.1.3)(https://www.R-project.org/) to extract the expression of the target gene PAQR4 in normal tissues and cancers. The differences were obtained by R package “plyr (1.8.7)” and “reshape2 (1.4.4)”. The PAQR4 expression activity between normal and tumor tissues was analyzed by the “GSEABase (1.56.0)” package and Gene Set Variation Analysis (GSVA).

### Clinical correlation analysis

The relations between PAQR4 expression and tumor stage, age, and gender were discussed. The analysis results were visualized by the “ggplubr (0.5.0)” package.

### PAQR4 expression and prognosis

We analyzed survival data downloaded from the UCSC Xena database and examined the overall survival (OS), disease-specific survival (DSS), disease-free survival (DFS), and progression-free survival (PFS) of PAQR4 in pan-cancer. Kaplan-Meier method was used for survival analyses by R package “survival (3.3-1)” and “survMiner (0.4.9)”. The relation between PAQR4 expression and survival in pan-cancer was also analyzed by Cox analysis and forest plots using the R packages “survival (3.3-1)” and “forestplot (2.0.1)”.

### Immune-related analysis

Immunological and stromal scores of tumor samples were obtained by R packages “estimate (1.0.13)” and “limma (3.50.3)” using the ESTIMATE method^25^. The results of immune cell infiltration in all tumor samples were obtained by the R package “limma”^26^. The Tumor-Immune System Interactions and Drug Bank (TISIDB) database (http://cis.hku.hk/TISIDB/) was used to study the relations between PAQR4 expression and immune stimulators, immune inhibitors, and major histocompatibility complex (MHC) molecules. It was also used to obtain the association of PAQR4 with immune subtypes.

### Tumor mutation burden and microsatellite instability

We analyzed the correlation of TREM2 expression with tumor mutation burden (TMB) and microsatellite instability (MSI) in pan-cancer using the R package “fmsb (0.7.3)”, and the results were presented as radar plots.

### Gene set enrichment analysis

Gene Ontology (GO) and Kyoto Encyclopedia of Genes and Genomes (KEGG) gene sets (c5.go.v7.5.1.symbols.gmt, c2.cp.kegg.v7.5.1.symbols.gmt) were downloaded through the Gene Set Enrichment Analysis (GSEA) website^27,28^ (http://www.gsea-msigdb.org/gsea/index.jsp). We used the R packages “limma (3.50.3)”, “org.Hs.eg.db (3.14.0)”, “clusterProfiler^29^ (4.2.2)”, and“enrichplot (1.14.2)” for functional analysis.

### Cell culture

Human bladder cancer cell lines (5637 and T24) and kidney clear cell cancer cell lines (CAKI1 and 786-O) were purchased from the Cell Resource Center Affiliated to the Chinese Academy of Medical Sciences, and cultured in RPMI-1640 medium (Biological Industries) supplemented with 10% fetal bovine serum (Biological Industries) and 1% penicillin-streptomycin (100 units/ml, Solarbio, Beijing, China). We cultured cells in a humidified incubator with 5% CO_2_ at 37 °C and changed the culture medium based on cell density.

### Quantitative real-time RT-PCR and siRNA molecular transfection

Total RNA was isolated and purified from clinical patient tissues and cancer cell lines using the Total RNA Kit (Omega Bio-Tek, USA) according to the manufacturer's instructions. The qRT-PCR assay was performed as instructed. The primers were designed as follows: PAQR4-F: TGCCCGCCTACTGGTATTTG, PAQR4-R: GCTCAGCTGCATGATCT. GAPDH-F:

GGAAGGTGAAGGTCGGAGTCA, GAPDH-R: GTCATTGATGGCAACAATATCCACT. PAQR4 was silenced using siRNA oligonucleotides (GenePharma, Suzhou, China), and the sequences were as follows: si1: GCAACUCCCACCAGAUCAUTT, AUGAUCUGGUGGGAGUUGCTT; si2: ACAACGAACUGGGCAACAUTT, AUGUUGCCCAGUUCGUUGUTT. All experiments were repeated at least three times. The studies involving human participants were reviewed and approved by the Medical Ethics Committee of the Second Hospital of Tianjin Medical University. The patients provided their written informed consent to participate in this study. All experiments were performed in accordance with relevant guidelines and regulations.

### Immunohistochemistry

We selected pathological wax blocks from patients with renal clear cell carcinoma and patients with bladder cancer and cut paraffin sections of cancer and paracancerous tissues. Tris-EDTA (Solarbio, Beijing, China) was used for antigen repair, Zhongshan Jinqiao immunohistochemistry kit PV-9000 was used for experiments, the anti-PAQR4 antibody (NOVUS, NBP2-83356) was diluted at 1:200, and DAB reagent (Zhongshan Jinqiao, ZLI-9018) was used for color development.

### Western blot assay

Total protein was extracted using RIPA buffer supplemented with PMSF. Protein concentration was measured using the BCA assay (Solarbio, Beijing, China). Proteins were separated using 10% SDS/PAGE gels and transferred to NC membranes. Cut the membrane before hybridization with antibodies during blotting according to the molecular weight of the target proteins. Incubate overnight with anti-PAQR4 antibody (NOVUS, NBP2-83356) and anti-GAPDH antibody (Proteintech) and detect the bound antibody with HRP-conjugated secondary antibody. ECL chemiluminescence kit (Solarbio, Beijing, China) was used to visualize the results. The image after cutting is shown in supplementary Fig. 5.

### Cell counting kit-8(CCK-8) assay, colony formation assay, and EdU assay

CCK-8 (CCK-8 solution: Solarbio, Beijing, China) was used to assess the viability of the cells. We seeded 2000 transfected cells in 96-well with 100 μl 10% FBS cell culture mediumand measured the optical density (OD) value after incubation at 37 °C for 3 hours. We detected the optical density value at 450 nm at the same time every day for four days. For colony formation assay, the transfected cells were seeded in 6-well plates with 300 cells per well supplemented with 2 mL 10% FBS cell culture medium and cultured for at least one week. The 6-well plates were fixed with 4% paraformaldehyde for 15 min and then stained with 0.1% crystal violet solution for 20 min. The EdU assay was also used to verify the proliferative ability of the cells. The transfected cells were incubated with EdU (Abbkine, China), and cell nuclei were stained with DAPI, and observed by fluorescence microscope. All experiments were repeated at least three times.

### Wound healing assay

Cells were inoculated in six-well plates, and when the cell fusion rate reached 70–80%, the cells were scribed using a pipette tip, washed three times with PBS, and photographed in the same field of view at 0h, 16h, and 32h, respectively.

### Transwell assay

Matrigel was melted at 4 °C, diluted with pre-chilled RPMI-1640 at a ratio of 1:3, and spread on the basement membrane of the chambers. Medium containing 10% fetal bovine serum was added to the bottom of 24-well plates, cells were starved for 12h in advance, added to transwell chambers, and then cultured in an incubator for 24h. The chambers were removed and the unpenetrated cells were wiped off with cotton swabs, fixed with paraformaldehyde for 20min, and stained with 0.1% crystalline violet solution for 10min. The chamber was placed under a microscope for observation after washing.

### Statistical analysis

All statistical analyses were processed by R software, and *, **, *** mentioned in the article represent *P-value* < 0.05, *P-value* < 0.01, and *P-value* < 0.001, respectively.

### Ethics declarations 

The studies involving human participants were reviewed and approved by the Medical Ethics Committee of the Second Hospital of Tianjin Medical University. The patients provided their written informed consent to participate in this study. All experiments were performed in accordance with relevant guidelines and regulations.

## Supplementary Information

Supplementary Information.

## Data Availability

Transcriptome data and related clinical data of 33 cancers were downloaded from TCGA through the UCSC Xena platform (https://xenabrowser.net/datapages/). Other data can be found in the HPA database (http://www.proteinatlas.org/), TISIDB database (http://cis.hku.hk/TISIDB/), and GSEA (http://www.gsea-msigdb.org/gsea/index.jsp). All data is publicly available.

## Abbreviations

ACC: Adrenocortical carcinoma
BLCA: Bladder urothelial carcinoma
BRCA: Breast invasive carcinoma
CESC: Cervical squamous cell carcinoma and endocervical adenocarcinoma
CHOL: Cholangiocarcinoma
COAD: Colon adenocarcinoma
DLBC: Lymphoid neoplasm diffuse large B-cell lymphoma
ESCA: Esophageal carcinoma
GBM: Glioblastoma multiforme
HNSC: Head and Neck squamous cell carcinoma
KICH: Kidney Chromophobe
KIRC: Kidney renal clear cell carcinoma
KIRP: Kidney renal papillary cell carcinoma
LAML: Acute myeloid leukemia
LGG: Brain lower grade glioma
LIHC: Liver hepatocellular carcinoma
LUAD: Lung adenocarcinoma
LUSC: Lung squamous cell carcinoma
MESO: Mesothelioma
OV: Ovarian serous cystadenocarcinoma
PAAD: Pancreatic adenocarcinoma
PCPG: Pheochromocytoma and paraganglioma
PRAD: Prostate adenocarcinoma
READ: Rectum adenocarcinoma
SARC: Sarcoma
SKCM: Skin Cutaneous Melanoma
STAD: Stomach adenocarcinoma
TGCT: Testicular germ cell tumors
THCA: Thyroid carcinoma
THYM: Thymoma
UCEC: Uterine corpus endometrial carcinoma
UCS: Uterine carcinosarcoma
UVM: Uveal melanoma
