# Supplementary material for: Pan-cancer analysis of the prognostic and immunological role of PAQR4
Source: Sci Rep. 2022 Dec 8;12:21268. doi: 10.1038/s41598-022-25220-3 (PMC9732355; doi:10.1038/s41598-022-25220-3)
Supplement: Supplementary file 1 — Supplementary Information. [file 41598_2022_25220_MOESM1_ESM.pdf]

Supplementary Material

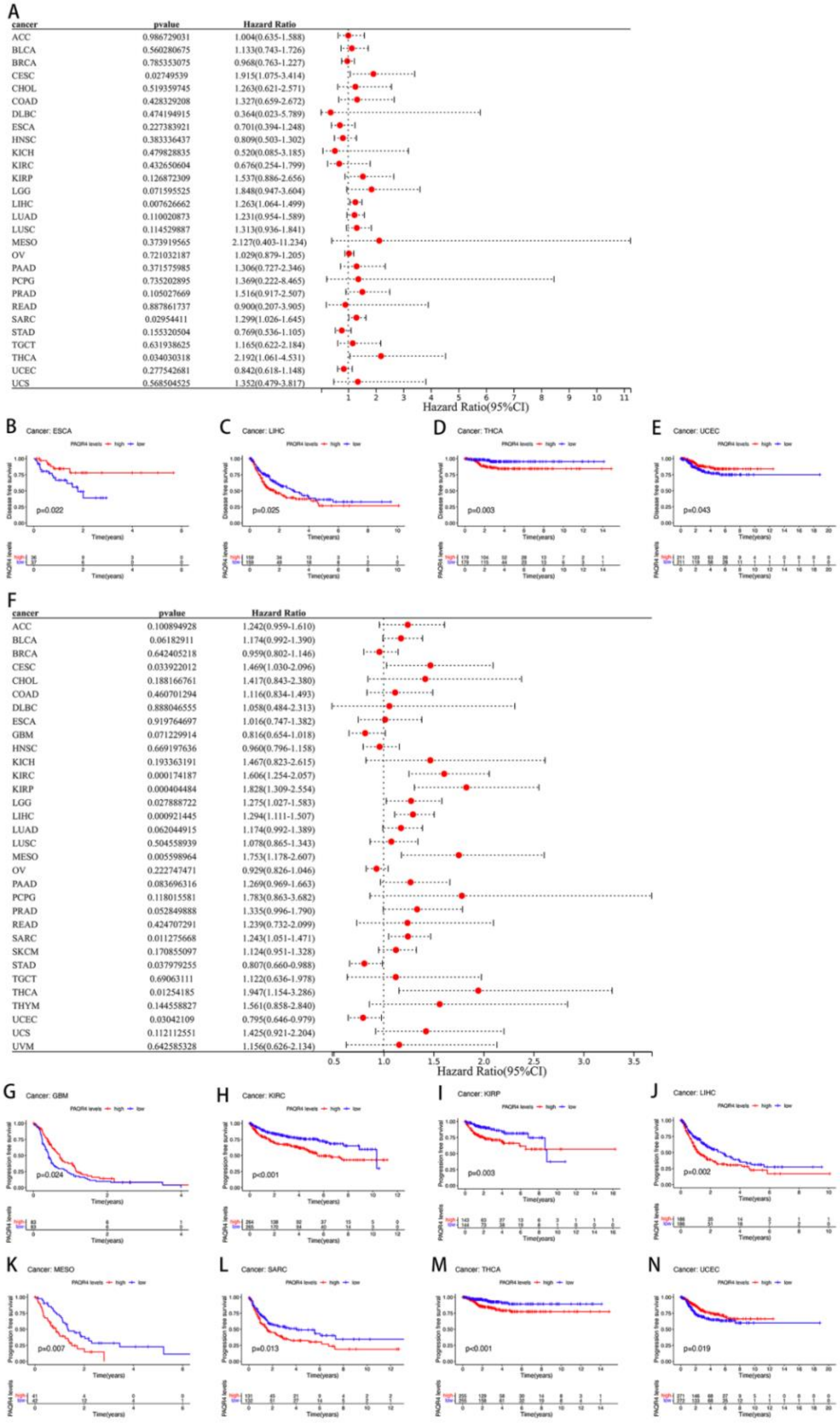

Supplementary Figure 1. Prognostic value of PAQR4 in pan-cancer. (A) Forest plot of the association between PAQR4 and disease-free survival (DFS) in 28 tumors. (B-E) Kaplan-Meier analysis of the association between PAQR4 expression and DFS in each tumor. (F) Forest plot of the association between PAQR4 and progression-free survival (PFS) in 32 tumors. (G-N) Kaplan-Meier analysis of the association between PAQR4 expression and PFS in each tumor.

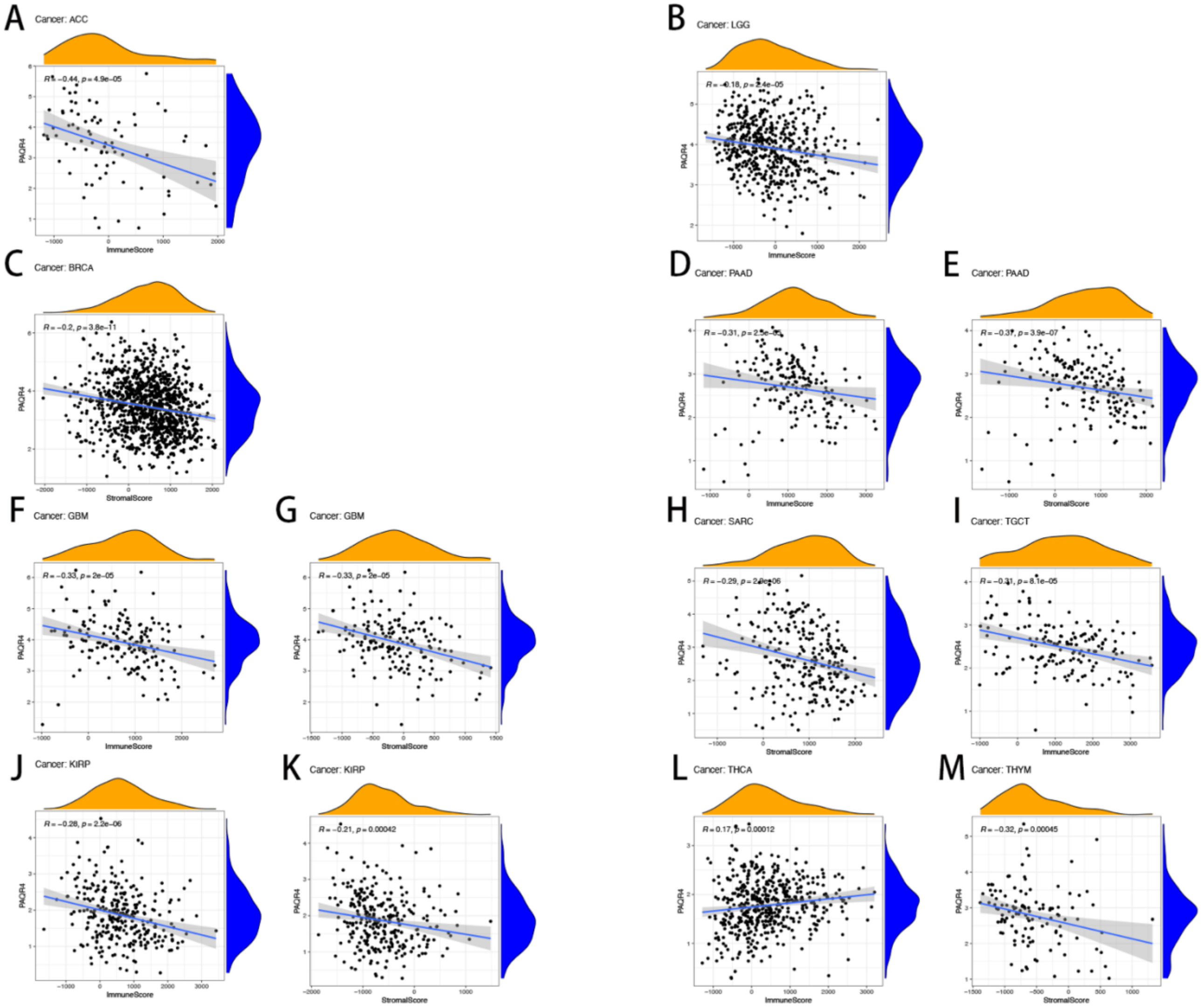

Supplementary Figure 2. Association of PAQR4 expression with immune score and stromal score in a variety of tumors.

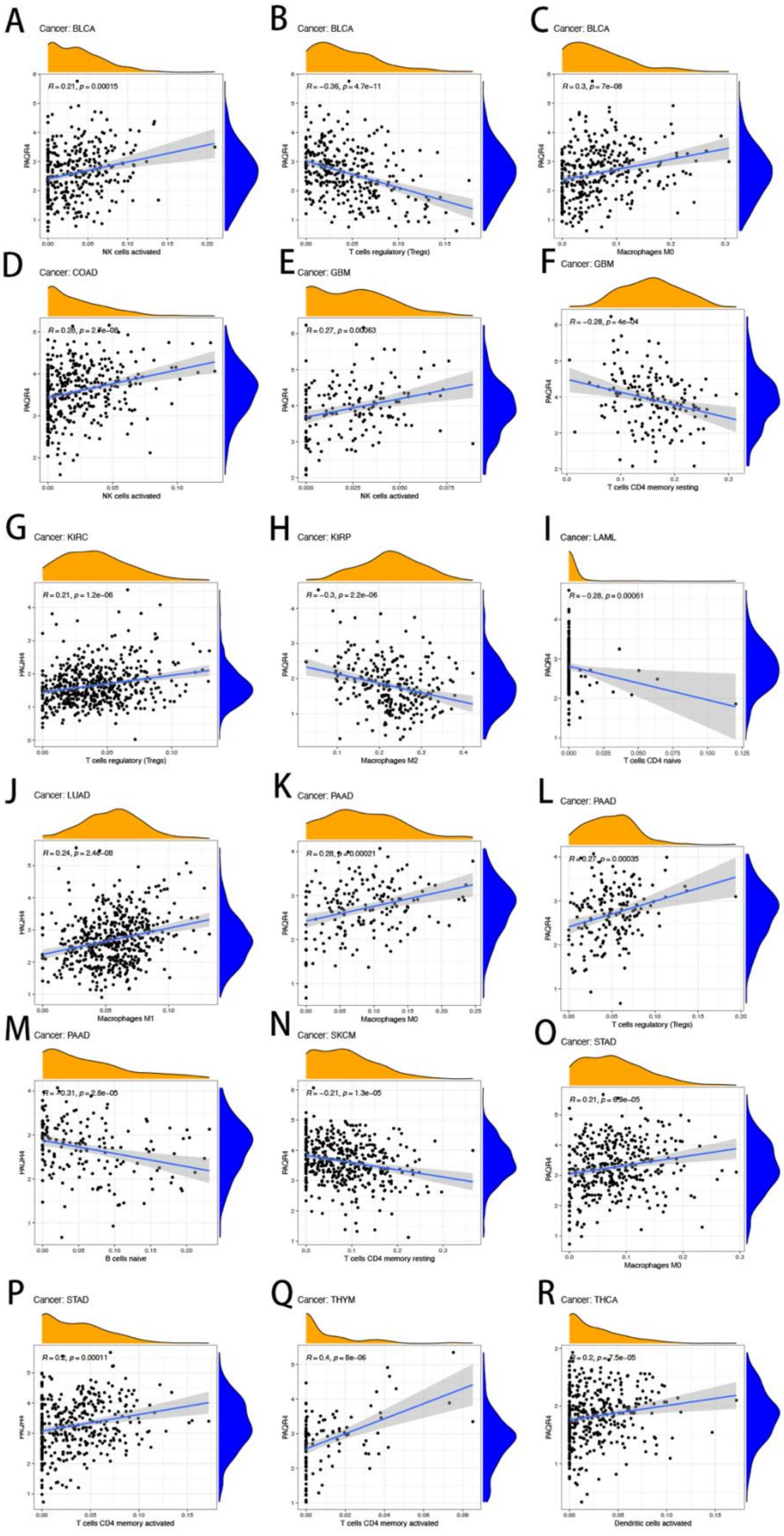
A
Cancer: BLCA
R = 0.21, p = 0.00015
PAQR4
NK cells activated
B
Cancer: BLCA
R = −0.36, p = 4.7e−11
PAQR4
T cells regulatory (Tregs)
C
Cancer: BLCA
R = 0.3, p = 7e−08
PAQR4
Macrophages M0
D
Cancer: COAD
PAQR4
NK cells activated
E
Cancer: GBM
PAQR4
NK cells activated
F
Cancer: GBM
R = −0.28, p = 4e−04
PAQR4
T cells CD4 memory resting
G
Cancer: KIRC
R = 0.21, p = 1.2e−06
T cells regulatory (Tregs)
H
Cancer: KIRP
R = −0.3, p = 2.2e−06
PAQR4
Macrophages M2
I
Cancer: LAML
R = −0.28, p = 0.00061
PAQR4
T cells CD4 naive
J
Cancer: LUAD
Macrophages M1
K
Cancer: PAAD
PAQR4
Macrophages M0
L
Cancer: PAAD
PAQR4
T cells regulatory (Tregs)
M
Cancer: PAAD
B cells naive
N
Cancer: SKCM
R = −0.21, p = 1.3e−05
PAQR4
T cells CD4 memory resting
O
Cancer: STAD
PAQR4
Macrophages M0
P
Cancer: STAD
T cells CD4 memory activated
Q
Cancer: THYM
R = 0.4, p = 8e−06
PAQR4
T cells CD4 memory activated
R
Cancer: THCA
PAQR4
Dendritic cells activated

Supplementary Figure 3. Association of PAQR4 expression with immune cell infiltration in a variety of tumors.

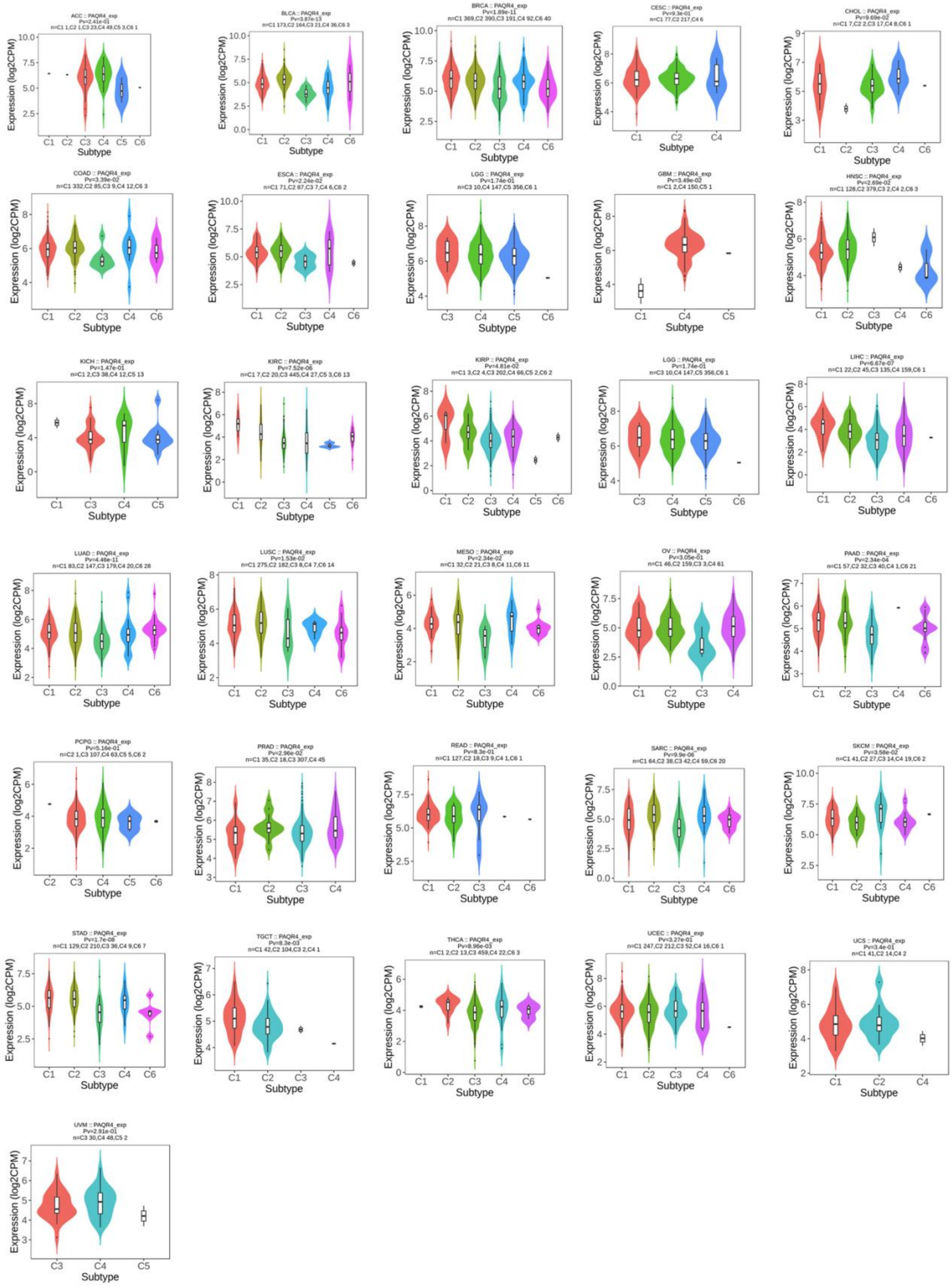
ACC :: PAQR4_exp
Pv=2.41e-01
n=C1 1,C2 1,C3 23,C4 49,C5 3,C6 1
BLCA :: PAQR4_exp
Pv=3.87e-13
n=C1 173,C2 164,C3 21,C4 36,C6 3
BRCA :: PAQR4_exp
Pv=1.89e-11
n=C1 369,C2 390,C3 191,C4 92,C6 40
CESC :: PAQR4_exp
Pv=9.3e-01
n=C1 77,C2 217,C4 6
CHOL :: PAQR4_exp
Pv=9.69e-02
n=C1 7,C2 2,C3 17,C4 8,C6 1
COAD :: PAQR4_exp
Pv=3.39e-02
n=C1 332,C2 85,C3 9,C4 12,C6 3
ESCA :: PAQR4_exp
Pv=2.24e-02
n=C1 71,C2 87,C3 7,C4 6,C6 2
LGG :: PAQR4_exp
Pv=1.74e-01
n=C3 10,C4 147,C5 356,C6 1
GBM :: PAQR4_exp
Pv=3.49e-02
n=C1 2,C4 150,C5 1
HNSC :: PAQR4_exp
Pv=2.69e-02
n=C1 128,C2 379,C3 2,C4 2,C6 3
KICH :: PAQR4_exp
Pv=1.47e-01
n=C1 2,C3 38,C4 12,C5 13
KIRC :: PAQR4_exp
Pv=7.52e-06
n=C1 7,C2 20,C3 445,C4 27,C5 3,C6 13
KIRP :: PAQR4_exp
Pv=4.81e-02
n=C1 3,C2 4,C3 202,C4 66,C5 2,C6 2
LGG :: PAQR4_exp
Pv=1.74e-01
n=C3 10,C4 147,C5 356,C6 1
LIHC :: PAQR4_exp
Pv=6.67e-07
n=C1 22,C2 45,C3 135,C4 159,C6 1
LUAD :: PAQR4_exp
Pv=4.46e-11
n=C1 83,C2 147,C3 179,C4 20,C6 28
LUSC :: PAQR4_exp
Pv=1.53e-02
n=C1 275,C2 182,C3 8,C4 7,C6 14
MESO :: PAQR4_exp
Pv=2.34e-01
n=C1 32,C2 21,C3 8,C4 11,C6 11
OV :: PAQR4_exp
Pv=3.05e-01
n=C1 46,C2 159,C3 3,C4 61
PAAD :: PAQR4_exp
Pv=2.34e-04
n=C1 57,C2 32,C3 40,C4 1,C6 21
PCPG :: PAQR4_exp
Pv=5.16e-01
n=C2 1,C3 107,C4 63,C5 5,C6 2
PRAD :: PAQR4_exp
Pv=2.96e-02
n=C1 35,C2 18,C3 307,C4 45
READ :: PAQR4_exp
Pv=8.3e-01
n=C1 127,C2 18,C3 9,C4 1,C6 1
SARC :: PAQR4_exp
Pv=9.9e-06
n=C1 64,C2 38,C3 42,C4 59,C6 20
SKCM :: PAQR4_exp
Pv=3.58e-02
n=C1 41,C2 27,C3 14,C4 19,C6 2
STAD :: PAQR4_exp
Pv=1.7e-08
n=C1 129,C2 210,C3 36,C4 9,C6 7
TGCT :: PAQR4_exp
Pv=8.3e-03
n=C1 42,C2 104,C3 2,C4 1
THCA :: PAQR4_exp
Pv=8.96e-03
n=C1 2,C2 13,C3 459,C4 22,C6 3
UCEC :: PAQR4_exp
Pv=3.27e-01
n=C1 247,C2 212,C3 52,C4 16,C6 1
UCS :: PAQR4_exp
Pv=3.4e-01
n=C1 41,C2 14,C4 2
UVM :: PAQR4_exp
Pv=2.91e-01
n=C3 30,C4 48,C5 2
Expression (log2CPM)
Subtype

Supplementary Figure 4. Relationship between PAQR4 expression and tumor immune subtypes in the TISIDB database.

A

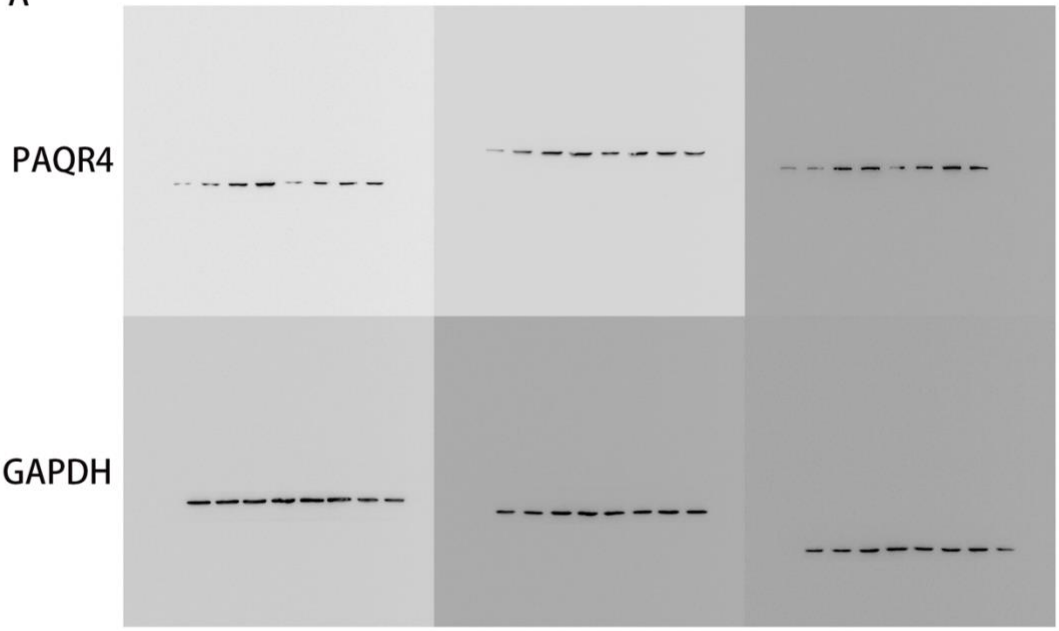

B

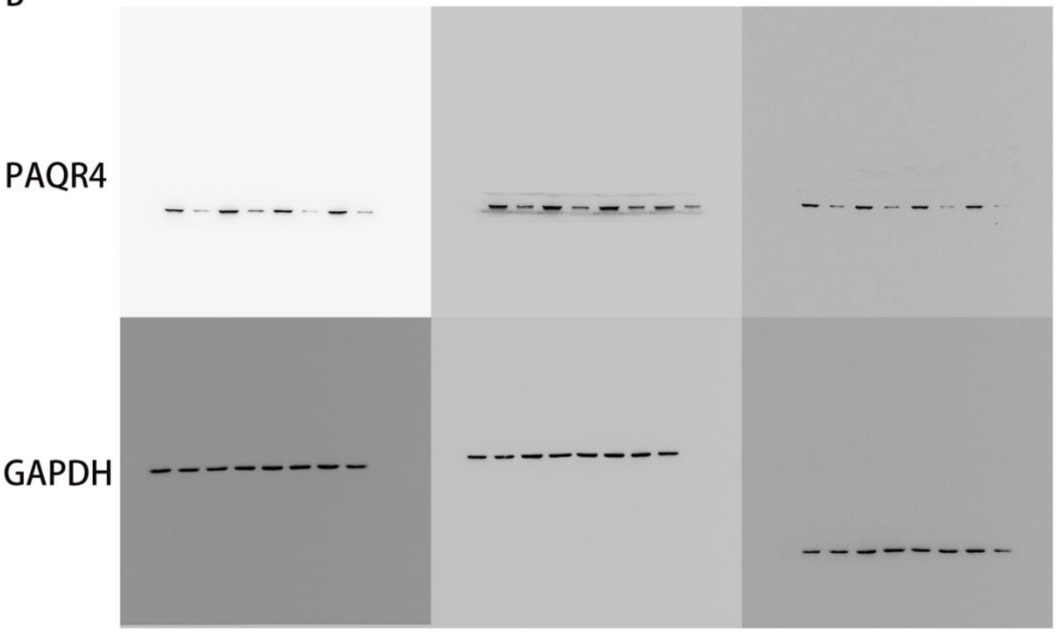

C

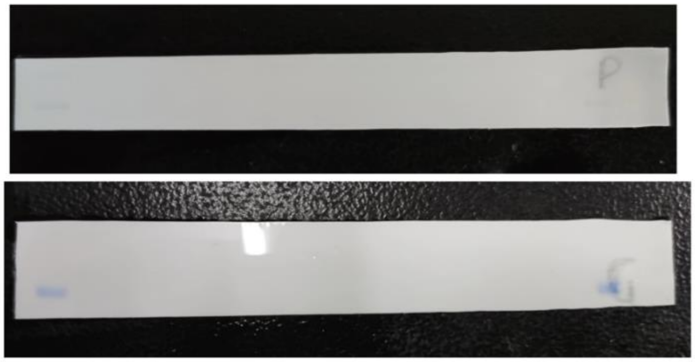

- **Supplementary Figure 5.** Complete results of Western blot. (A) Sample order: CAKI, 786-O, 5637, T24, CAKI, 786-O, 5637, T24. (B) Sample order: 786-O siNC, 786-O si1, T24 siNC, T24 si1, 786-O siNC, 786-O si1, T24 siNC, T24 si1. (C) Example of blots after cutting.
